# Gauge Blocks – A Zombie Technology

**DOI:** 10.6028/jres.113.013

**Published:** 2008-06-01

**Authors:** Ted Doiron

**Affiliations:** National Institute of Standards and Technology, Gaithersburg, MD 20899

**Keywords:** end standard, gage blocks, process control, stability, technical obsolescence, thermal expansion

## Abstract

Gauge blocks have been the primary method for disseminating length traceability for over 100 years. Their longevity was based on two things: the relatively low cost of delivering very high accuracy to users, and the technical limitation that the range of high precision gauging systems was very small. While the first reason is still true, the second factor is being displaced by changes in measurement technology since the 1980s. New long range sensors do not require master gauges that are nearly the same length as the part being inspected, and thus one of the primary attributes of gauge blocks, wringing stacks to match the part, is no longer needed. Relaxing the requirement that gauges wring presents an opportunity to develop new types of end standards that would increase the accuracy and usefulness of gauging systems.

## 1. Introduction

In their 1907 book *Accurate Tool Work*, C. L. Goodrich and F. A. Stanley [1] describe many of the standard measurement tools such as master plates, buttons, disks, size blocks, etc. They also complain that “It is really surprising that, considering the great development to which these methods have been carried, permanent literature relating to them scarcely exists.” Now 100 years later the situation is better in that there are numerous books describing simple measurement methods, but few go past simple measuring tools and the documentation of 19th century methods for quality control is not much better than it was in 1907.

Despite the difficulty, it is possible to give a rough outline of how dimensional measurement developed in the industrial revolution, and this development will bring us to the technical and economic forces that produced the gauge block, and the technical and economic forces that are slowly causing their demise [2].

While gauge blocks are not quite dead yet, they are also not quite alive. Gauge blocks were invented and used because they met the technical problems of their time in the most efficient manner. While measurement technology has seen a revolution in the last 30 years, gauge blocks have not changed at all. While gauge blocks should be dead and gone, they persist. The dictionary (dictionary.com) defines a zombie (in voodoo) as:

the body of a dead person given the semblance of life, but mute and will-less, by a supernatural force, usually for some evil purpose.

By analogy, a Zombie Technology could be defined as:

A dead technology given a semblance of life, but awkward and inefficient, by a supernatural force (human inertia) usually with evil (costly) effects.

At NIST the Engineering Metrology Group still calibrates a lot of gauge blocks, around 4,500 a year, but the average number in a customer order has been dropping for some years. We increasingly get small sets of two to four blocks, a trend that will probably continue. In this paper I try to provide an overview of the causes for this development and some of the opportunities for new and better measurements.

## 2. Rise of Interchangeable Parts (American System, first Industrial Revolution)

**Fig. 1:**
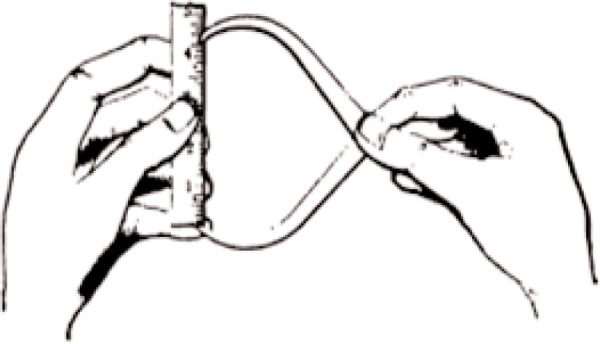
Early 19th century measurements were primarily simple comparisons to a rule or master part.

The measurement needs of the industrial revolution began with the need to make interchangeable parts. The initial efforts were primarily in weaponry, with Eli Whitney and the Colt companies, but at the beginning of the 19th century a factory was not what we would consider a factory today, and the idea of mass production had not taken hold. A factory was usually a shop of 50 or fewer craftsmen who essentially made all of the parts of a product and assembled the final product. It was much like the older craftsman system except that the parts were made to match a model product. With the use of jigs to aid in making repeatable rough parts, the rest of the work was done by hand. The measurement demands could be met with simple comparison of master parts or rules with a simple caliper as in Fig. 1. The use of model sets of parts was in common use by the 1820s. Making the parts to tolerances of a few tenths of a millimeter was adequate to give interchangeable parts for guns, although the actual quality of the guns was no better, and often worse, than available from the old craftsman system [3].

By the middle of the 19th century the manufacturing systems technology was rapidly changing. The development of machine tools was beginning, the organization of factories was changing, and the demands for speed and accuracy were increasing. These challenges were met with the use of limit gauges for critical dimensions.

## 3. Rise of the Machine Tool

**Fig. 2:**
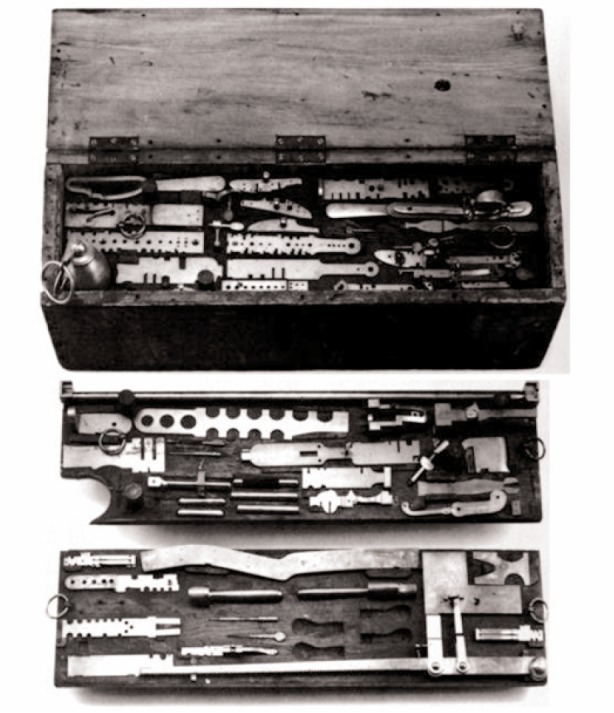
One complete set of gauges used in the manufacture of a single model pistol.

In the middle of the 19th century there were two major trends. The first was the continued development of production methods for products with interchangeable parts. The number of dimensions that were controlled was rising, and the system of limit gauges was gaining in sophistication. Figure 2 shows a complete set of gauges for making a single model gun from the National Museum of American History [4]. Using these gauges was much like the GO/NO GO limit gauges still in use today. There also was a hierarchy of these gauges with a master set of gauges, inspection gauges and working gauges for the shop. These gauges were regularly compared because of the wear from every day use. There were, however, a lot of gauges to maintain. For every working gauge at the shop level there was both a reference gauge to check it for wear in the tool room and a master gauge.

While the methods for producing interchangeable parts were being perfected, there was an ongoing revolution in machine tools [5]. The speed of machine tool development in the 19th century was astonishing, outpacing the development of measurement technology. The organization of the factory was also changing. Newer manufacturing processes required more expertise from the workers. The trend was for more specialization in the workforce with supervisors to keep the workers’ efforts synchronized. Overall, the division of labor changes and the efficiency and accuracy of the gauging system made the factory more efficient and raised the quality of the product significantly.

The increasing control of making parts resulted in tighter tolerances and further developments in measuring technology. The need for more accurate measurements led to the development of measuring instruments such as vernier calipers, micrometers and indicator gauges. The earliest micrometer was probably that of James Watt in the 18th century. Maudsley had working forms of the vernier caliper in the early 19th century, and Whitworth had his “millionth” micrometer in the 1860s. None of these technologies spread to industry because they were slower than simple calipers and their accuracy capabilities were beyond the needs of early factories.

This began to change in the second half of the 19th century as the second Industrial Revolution began. The variation between operators using simple calipers was small enough to make working parts, but as design demands and machine tool performance rose, new measuring systems were needed. By the 1860s, efforts were beginning to industrialize the production of micrometer and vernier calipers, as well as other high precision instruments. Mass production of these tools made them reasonably inexpensive, and satisfied the need for more accurate measurements, particularly in the tool room, where working gauges were checked against the part drawings. Shop floor measurements were still dominated by limit gauges because of their speed, but the accuracy of the limit gauges was constantly rising. Overall, however, the number of gauges was growing quickly, and the three levels of gauges were difficult and expensive to maintain.

**Fig. 3:**
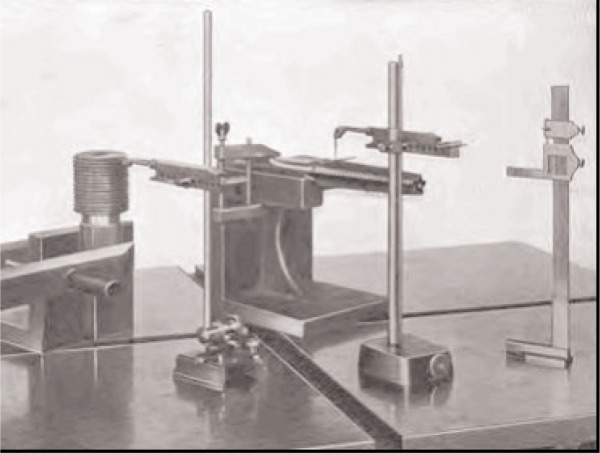
Surface plate with vernier height gauge and mechanical indicator gauge.

Another development was the use of very flat reference surfaces, surface plates. The earliest recorded use of surface plates was again Maudsley, and again, the method was not adopted by industry; it was not the time. The redevelopment of the method by Whitworth 50 years later was the right time for industry adoption. The method used three plates, which were intercompared and the high portions scraped away until all three were flat. The flat surface, along with vernier height gauges and limit gauges, came together to form the basic surface plate metrology still in use in most factories. Figure 3 shows a typical use of the surface plate as a reference surface, a vernier height reference and some mechanical indicator gauges to check part dimensions.

Besides the advances in technology (machine and measuring tools), the rise in specialization led to the need for communication between all of the scattered parts of the company’s factory or factories. The answer was the development of detailed scale drawings with nominal sizes. The changeover from master parts to drawings was not as easy as you might expect [6].

The rise of standardized mechanical drawings began in the early 19th century with the first manuals of drawing, but it was not until the 1840s that drawing made any real penetration into the curriculum of the common schools. The idea of making things without an accurate drawing seems strange to us today, but the ability to look at scale drawings and visualize how to make and assemble the parts is a specific skill that had to be developed. The earliest inventors had a very physical intuition of mechanisms, and as drawings came to be a significant tool in industry, many of them simply could not make the transition. The educational effort of industry, as well as industry leaders’ influence on public and private school curriculum, is an interesting story in itself. While the first texts and manuals appeared in the first years of the 19th century, the acceptance of drawing as a worthy subject in schools never received wide acceptance. In the end, teaching of the formalized drawing systems fell to the engineering schools and industry training systems.

The combination of mechanical drawings, standard reference planes, and accurate measuring instruments formed the core of the quality system for over a century.

## 4. Rise of Gauge Blocks

The gauge block was the right invention at the right time. Their usefulness is based on wringing. When two blocks of metal with hard flat surfaces are slid together, the surfaces wring to each other, that is, there is a large force holding them together, and the composite length is the sum of the two individual pieces within a fraction of a micrometer. The fact that two parallel steel surfaces would “wring” together was a well known effect. In 1856, Whitworth read a paper describing the effect before the Institute of Mechanical Engineers in Glasgow [7]. In 1875 Dr. Tyndall described experiments that showed the surfaces have this property even in a vacuum, and in some cases the force between the plates was 30 times that of gravity [8].

**Fig. 4a:**
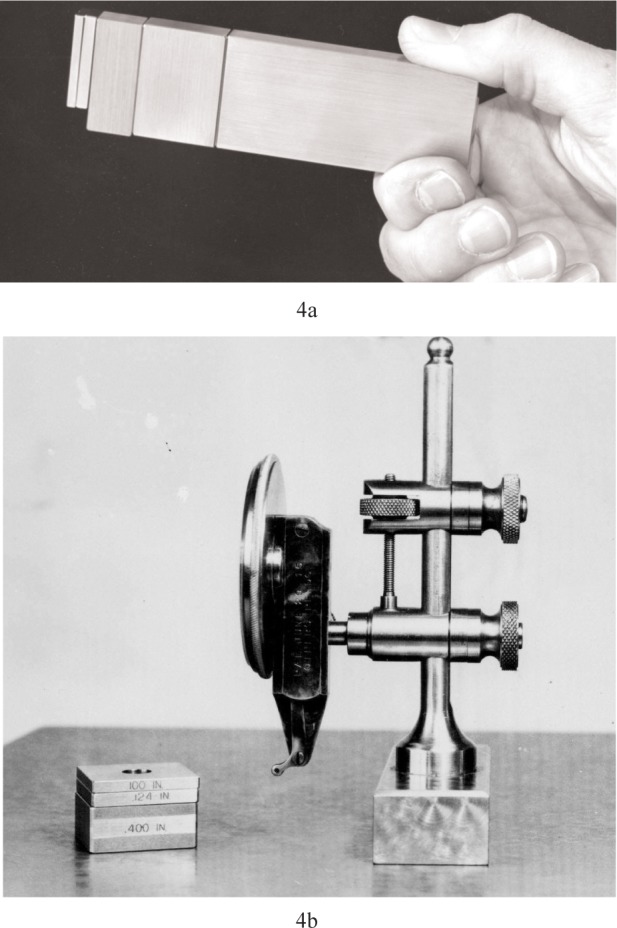
A stack of gage blocks wrung together will make a mechanically stable composite gage of any size to the nearest micrometer. This stack can then be used as a master length to set small range sensors, as shown in Fig. 4b.

C. E. Johannson was an employee of a rifle factory in Sweden when he had an idea that could reduce the large number of individual length standards in the factory [9]. His idea, first formulated in 1896, was that a small set of gauges that could be combined to form composite gauges could reduce the number of gauges needed in the shop. For example, gages of sizes 1 mm, 2 mm, 4 mm, and 8 mm could be wrung in any combination, and all of the millimeter sizes from 1 mm to 15 mm could be made from only these four gauges. Examples are shown in Fig. 4.

With the proper use of accessories, a stack of blocks could be used as an internal or external limit gauge for nearly any dimension with better accuracy than most fixed limit gauges in use. The reduction in the number of gauges that had to be maintained made them both convenient and cost effective. In the United States, they had become so important that when the gage block supply was cut off during World War I, the government relieved the problem by having the Bureau of Standards (now NIST) develop a method to mass produce blocks for industry. In the auto industry they became so important that in the 1920s Henry Ford bought the Johansson factory in New York and moved it to Michigan. Gage blocks were the main source of traceability for dimensional measurements for a century.

## 5. Rise of High Precision Sensors

As industry progressed, tolerances became tighter, and higher precision was needed in measurement. There were two main branches of development: mechanical and optical. The continued development of older technology produced a number of very sensitive indicators such as the Mikrokator (1938), the Sheffield Visual Gauge Comparator, and various forms of air gauging. Descendents of these systems are still in use. Optical levers and the Mikrokator were delicate instruments and were primarily used in calibration labs. Electronic gauging began with the invention of the Linear Voltage Differential Transformer (LVDT) in 1946, and the combination of ruggedness and precision made it the dominant technology for many years. All of these systems, while providing resolutions of 0.1 μm or better, suffered because of their very narrow ranges. Most of these technologies had a typical accuracy of 1 % of their range, and for the most demanding gauging required a master length that was accurate to at most a few micrometers. For most of the 20th century there was only one technology that could economically meet these requirements for a master gauge length: gauge blocks.

By the middle of the 20th century, gauge blocks began to evolve in response to the increased accuracy requirements and their widespread use outside of the controlled environment of the calibration lab. New sizes with smaller differences in nominal length were needed for use with new, more precise indicators. The stability of steel gauge blocks was studied in great detail in the years around 1960, and heat treatments were found that produced very stable gauges with less than one part in 10^6^ length change per year. Over the next few decades materials were introduced that had other important properties, such as enhanced hardness to reduce wear. Currently, there are a very large number of different sizes, materials, and thermal expansion properties in use, and maintaining high accuracy for these widely varying gauges is increasingly difficult.

## 6. Rise of Long Range Sensors

As high throughput systems were being perfected, two technologies to replace gauge block sets had begun. The accuracy and range of laser interferometer systems became indispensable to very high accuracy measurements, they were very expensive and were generally confined to National Metrology Institutes and state-of-the-art industrial laboratories. While the costs associated with interferometers have come down, the development of linear scale systems has surpassed them for most measuring machines. While both systems are very interesting, both have been well documented and are familiar to most metrologists [10].

The important point is that the linear scale has the precision and accuracy needed to supplant the LVDT as the primary dimensional sensor, and has nearly any range needed. While the most precise systems still measure displacement, only one master length is needed to set the zero length point. Laser systems need two master lengths to set the zero and index of refraction correction to the wavelength, and most scale based machine users use a second length as a process check. Many NIST customers have begun to use gauge block sets of two to six blocks, two blocks of each of three materials. The use of different materials is to reduce the uncertainty from thermal expansion and mechanical deformation.

Nearly all calibration labs have the resources to buy a scale based micrometer with 300 mm or more range. Most already have such units, and most also have standard gauge block sets. The constant quest for efficiency and higher accuracy and lower costs is driving gauge blocks from most labs for the following reasons: First, the new equipment is more efficient and flexible. These instruments are for end standards, rings and cylinders. To wring up a stack of gauge blocks and wait for thermal equilibrium is slow, and slow costs money. The rising acceptance of ceramic gauges with only about half of the thermal diffusivity of steel increases the wait. While the system can be speeded up with fans [11], the process is still slow and unnecessary. Secondly, cost savings are possible. The accuracy/cost trade-offs are interesting. Obviously, costs are reduced if the company has fewer gauges calibrated. The standard metric set of gauge blocks has over 100 blocks. Even though gauge block calibration prices are very low, the sheer number makes a set expensive. But new measuring machines are also expensive and the cost recovery takes time.

Some companies have invested some of the cost savings by having the few gauges calibrated by higher accuracy suppliers. While NMI prices are very high, the cost of two to four blocks is still less than the cost of the full set at the lower commercial price. The lab saves some money and now has master gauges with the lowest available uncertainty. This can change the laboratory uncertainty, and with laboratory accreditation and publication of a lab’s best measurement capability, it may provide a marketing advantage.

Another set of our customers have changed their master gauges from gauge blocks to gauges of matched geometry. Generally, comparing rings, cylinders and spheres is more accurate when the master gauge is as much like the test gauge as possible. This trend is most evident in ring gauges, where our yearly calibration load has risen from 10 per year to well over 100 per year.

## 7. The Rise of End Standards

Gauge block sets, as we currently know them, will slowly be abandoned. The process has started and should be encouraged. The reason for the encouragement is that the definition of a gauge block carries a number of requirements for the material, processing and geometry that might be abandoned to our benefit. If a lab needs only a few length standards, should they be gauge blocks? To answer this question we will have to explore the characteristics required for an end standard to be a gauge block.

### 7.1 Wringability

The primary attribute of gauge blocks is that they wring together. The ability to slide two or more length standards together to get a new longer standard with an uncertainty of only a few tenths of a micrometer made gauge blocks indispensable for practical high accuracy measurements. But wringability also brings with it severe restraints on materials. The material needs to have a very hard stable surface, like hardened tool steel or various ceramics. Aluminum, with its oxide layer for example, will not wring. In the early 20th century most machine parts were steel; railroad equipment, automobiles, ships as well as most tools and appliances were based on steel. Today there is a wide span of materials like polymers, aluminum, super alloys and ceramics, while steel is an ever shrinking part of our products. The tightening dimensional specifications, coupled with the difference in thermal expansion properties between gauge blocks and modern materials, is a growing problem. If we drop the requirement of wringing, we can make end standards out of a much broader range of materials.

### 7.2 Stability

**Fig. 5:**
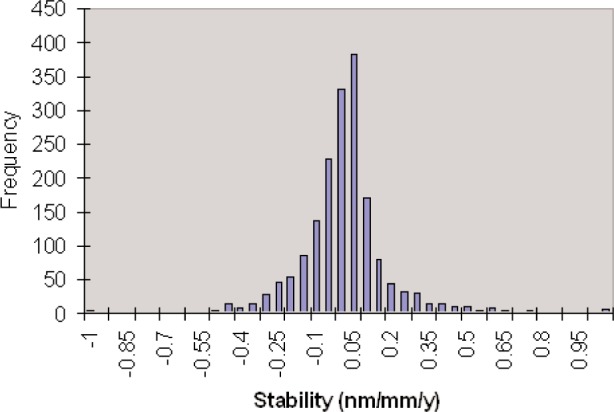
Stability of steel and chrome carbide gauge blocks.

Metrologists want gauges that stay the same length, or at least to some fraction of their length uncertainty, for a year or more. For high accuracy blocks in grades K and 0, the specification for relative stability is 0.25 × 10^−6^ per year. Finding a process to produce stable hardened tool steel was a long and difficult effort. At NIST in the 1950s there was a decade long project that developed the heat treatments to stabilize AISI 52100 steel. Currently the stability of gauge blocks in general is quite good. I compiled the calibration histories of about 3,000 gauge blocks that had been measured five times or more over at least 10 years and analyzed the variation in length over time [12]. Nearly all of the blocks were steel or chrome carbide, because we have not had customers with ceramic sets long enough to get good statistics. Early data show that new materials may not be as stable as steel and chrome carbide, but our calibration history does not have enough data to say definitively. The current data do show that they are generally stable enough to meet the standard. Figure 5 shows a histogram of the stabilities of these blocks.

The data on materials other than very low thermal expansion materials is unfortunately very sparse. Some measurements in our laboratory in the late 1980s on copper and copper alloys were discouraging. Samples of copper, brass, beryllium copper and tellurium copper were measured over time, and the results were all in the range of (1 to 5) × 10^−6^/y [13]. In the early 1990s NIST had a small program to measure the stability of some lightweight aerospace materials. There were twelve blocks made for each material, six nominally 50 mm long and six nominally 6 mm long. The data were compared to eliminate any changes from subsurface damage that occurs during the finishing process. Blocks were made from two types of aluminum (6061 and 5086 with two different heat treatments), two forms of silicon carbide (reaction bonded and chemical vapor deposition), and beryllium. The early data showed promise. Recent measurements after a hiatus of 16 years show that they have done surprisingly well, although the reaction bonded silicon carbide results are very strange. The small 6 mm samples shrank by nearly 2 % over the 15 years, while the 50 mm samples shrank slightly. While these results are not as good as current gauge blocks, with modern instrumentation and computers, moderate but linear stability may be adequate. If the length at one time and the change of length over time are known, the current length can be calculated.

The major problem with our method is that it takes so long to measure dimensional stability. Small gauge block sizes were used because our mechanical comparison system was our most reproducible measuring system. The length 50 mm was chosen as a trade off of higher sensitivity with longer blocks and larger variability from thermal effects. For this size the long term reproducibility was nearly 20 nm (1σ). The reproducibility to length ratio is 0.4 × 10^−6^.

This has changed since we moved our high accuracy CMM (Coordinate Measuring Machine) to its new environment in the Advanced Metrology Laboratory. The temperature stability is excellent in this lab space, and the length dependence of the reproducibility is remarkably small. This allows us to measure very long standards while retaining high precision. Control charts used for statistical process control on step gauges and end standards show a long term reproducibility of only 50 nm on 1 m measurements. The reproducibility to length ratio is only 0.05 × 10^−6^, a considerable improvement. The stability of 1 meter end standards could be measured to an accuracy of 1 part in 10^6^ in only a few months.

### 7.3 Length

**Fig. 6:**
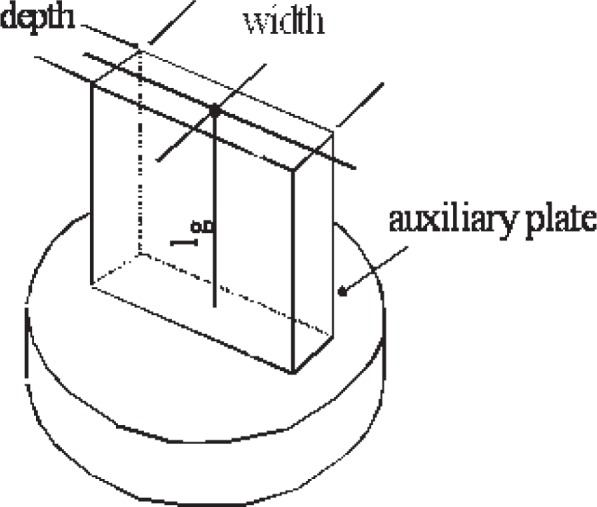
ISO 3650 defines the central length as the distance from the center to the plane surface of an auxiliary plate wrung to the bottom of the block.

The definition of length for a gauge block is unique among dimensional gauges because it is includes the “effect of one end wringing” [14]. The basic logic is this: Each block surface is not perfectly flat. The standard requires flatness better than 50 nm on Grades K and 0, and 100 nm on grades 1 and 2. When two blocks are wrung together, these wavy surfaces press together with some distortion of both surfaces. If the surfaces were perfect, the length of the combination would be the sum of the lengths of the two blocks, but with the varying geometry this is not true. If we could put this deformation into the definition of the block length, this new length for each block would add up properly. Having each block have a different definition of length for every other block it is wrung to is impractical, so the standard simulates the deformation correction by wringing the block to a very flat surface and defining the central length as the distance from the center of the top surface to the plane on which it is wrung, shown by Fig. 6.

When we use a gauge block or stack to check a measuring machine, we are assuming that the known length is the point to point distance between the centers of the two faces. Suppose we call the point to point length of one gauge L_1_, and the length of the second gauge L_2_. When measured interferometrically, we get this length modified by the effects of one end wringing. By putting the effect of an end wringing in the definition of length of each block, the length of a stack of blocks will automatically have some compensation for the wrings.

**Fig. 7:**
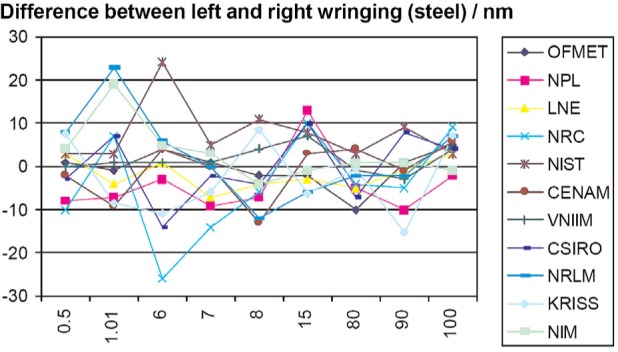
Difference in length between top down and bottom down calibration of gauge blocks on steel platens.

This might even be true, at least at the 20 nm level or so. The CCL (Consultative Committee for Length) K1 comparison on gauge block interferometry reported the lengths found with each end of each block wrung down [15]. The results from the report are shown in Fig. 7 (acronyms explained in [16]).

The pooled standard deviation for steel and tungsten carbide was 7 nm. This is nearly equal to the standard uncertainties reported by the participants.

**Fig. 8:**
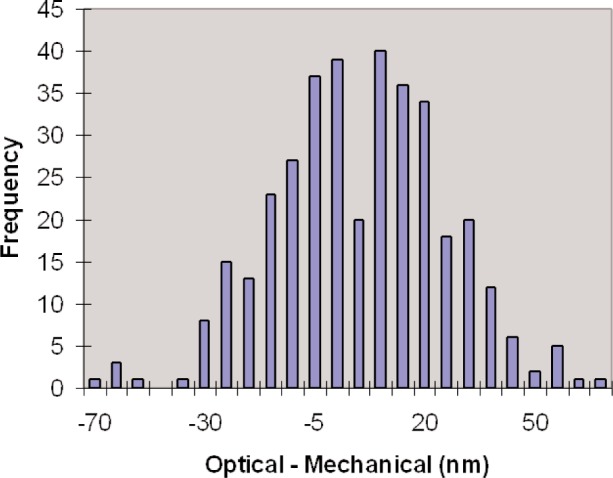
The histogram shows the distribution of the differences between the interferometric and mechanical lengths of 361 gauge block pairs. The standard deviation of the distribution is 17 nm.

A second estimate of this effect is the difference between the interferometric and mechanical lengths of gauge blocks. At NIST most customer calibrations are performed by mechanical comparison, and in each calibration a steel master and chrome carbide master are measured against the customer block. Since the defined length of each block is from interferometry, and we make over 4,000 comparisons between the two master blocks, the differences are not only visible but are a considerable nuisance. As the comparator tips are worn flat, the deformation between the contact and the blocks changes, and the correction between steel and chrome carbide slowly changes. After a few months the contacts have very small flats at the contact point, and the deformation correction becomes very stable at between 10 nm and 20 nm. As we are only interested in the range of the interferometric and mechanical length differences, the constant offset is not important. Figure 8 shows a histogram of the length differences on 361 pairs of master gauge blocks.

It is apparent from these data that the current definition of length for gauge blocks is a sizable component in the measurement uncertainty when they are used as end standards. I believe it is necessary to have a new type of standard gauge called the end standard. The most important requirement in the standard will be that the length will be defined as the point to point distance from the centers (or other reference points) of each gauging face. Another important specification is the squareness between the side of the gauge and the face of the gauge to reduce the possibility of cosine errors to a small fraction of the calibrated length uncertainty. The current tolerance in ISO 3650 is adequate for this, but the tolerance for square blocks in the United States is not. ASME B89.1.9 allows squareness errors up to 5 arc minutes, which will produce cosine errors as large as 10^−6^ times the length of the gauge. Other properties might be defined in the standard, but only if they provide some value such as economies of scale that would lower their costs or reduced alignment time.

## 8. Rise of the Solid Thermometer

An example of what opportunities might available from these new end standards would be the development of a totally solid thermometer. Most dimensional metrologists have noted at one time or another that a 500 mm gauge block would make a very good thermometer. The primary problem in using one long end standard of known coefficient of thermal expansion and length is that you are limited by the one-dimensional accuracy of the CMM.

Suppose we use two different end standards of very different thermal expansion coefficients, perhaps Zerodur^1^ and aluminum. Aluminum is a very high CTE (Coefficient of Thermal Expansion) material, perhaps the highest among materials of known stability. With the end standards mounted next to each other, the CMM will only compare the lengths, and the uncertainty in the length difference between the bars is basically limited by the one-dimensional repeatability of the machine. This is often very good, below 0.2 μm. Jim Salsbury presented this system of using two long end standards on a CMM, and it showed definite promise [17].

The CTE of Zerodur is negligible compared to the CTE of aluminum, 24 × 10^−6^/°C. If the one-dimensional repeatability of the CMM is 0.2 μm at each end, we can measure the difference in lengths to about 0.3 μm. If the standards are 500 mm long, the differential thermal expansion will be 12 μm/°C. The resolution of 0.3 μm converted to temperature is about 0.025 °C. The thermometer draws no power, there are no electronics to calibrate, and once the stability is known, there is little need for recalibration.

The second useful property of the Solid Thermometer is that it can serve as a check standard for the CMM. In the new edition of ISO 17025, a paragraph was added to the measurement assurance requirements (5.9.2):

5.9.2 Quality control data shall be analyzed and, where they are found to be outside predefined criteria, planned actions shall be taken to correct the problem and to prevent incorrect results from being reported.

This new language virtually requires some level of statistical process control. With the Solid Thermometer, the control data will be collected every time the temperature is measured.

## 9. Conclusion

Changes in technology force changes in other technology. Gauge blocks filled a critical need for inexpensive and flexible length gauges for over a century, a remarkably long period. The advent of high precision, long range measuring technology is changing the way gauge blocks are used, and we have an opportunity to change how we think about gauge blocks. Since old measuring technologies never completely die, we need to think about the properties for a new type of gauge, the end standard.

## Figures and Tables

**Table 1:** Stability of some low density materials

	nm/mm per year
5086 (Treatment 1)	−0.52
5086 (Treatment 2)	−0.49
6061 (Treatment 1)	−0.48
6061 (Treatment 2)	−0.46
SiC-CVD	0.05
Beryllium (I70-H)	−0.10

## Footnotes

1 Certain commercial equipment, instruments, or materials are identified in this paper to foster understanding. Such identification does not imply recommendation or endorsement by the National Institute of Standards and Technology, nor does it imply that the materials or equipment identified are necessarily the best available for the purpose.

## References

[1] Goodrich CL, Stanley FA (1907). Accurate Tool Work.

[2] Jaikumar Ramchandran (2005). From Filing Fitting to Flexible Manufacturing: A Study in the Evolution of Process Control, Foundations and Trends in Technology. Information and Operations Management.

[3] Van Dervort William H (1903). Modern Machine Shop Tools.

[4] Hounshell David A (1984). From the American system to mass production, 1800–1932.

[b5-v113.n03.a04] 5For many years the Smithsonian Museum in Washington DC had a large display of items from the 1876 Centennial Exposition. The largest part of the display was of machine tools and nearly every current machine tool has a recognizable progenitor in this display.

[6] Stevens (1995). The Grammar of the Machine.

[7] Durfee WF (1893). The History and Modern Development on the Art of Interchangeable Construction in Mechanism. Transactions of the Society of Mechanical Engineers.

[8] Goodeve TM, Shelley CPB (1877). The Whitworth Measuring Machine.

[9] Althin TKW, Johansson CE (1948).

[10] Kunzmann H, Pfeifer T, Flügge J (1993). Scales vs. Laser Interferometers, Performance and Comparison of Two Measuring Systems. Annals of the CIRP.

[11] Doiron T, McLaughlin D, Schneider A (2007). Use of Air Showers to Reduce Soaking Time for High Precision Dimensional Measurements.

[12] Doiron Ted, Schneider Anne (2008). Dimensional Stability of Some Dimensional Gauges. submitted to Measurement Science Conference.

[13] Doiron Ted, Stoup John, Snoots Patricia, Chaconas Grace (1990). Measuring the Stability of Three Copper Alloys, SPIE Volume 1335. Dimensional Stability.

[14] ISO 3650:1998(E) (1998).

[15] Thalmann Ruedi (2001). CCL Key Comparison CCL-K1: Calibration of gauge blocks by interferometry - Final report.

[b16-v113.n03.a04] 16Acronyms: Swiss Federal Office of Metrology (OFMET), National Physical Laboratory (NPL), Laboratoire national de métrologie et d’essais (LNE), National Research Council Canada (NRC), National Institute of Standards and Technology (NIST), Centro Nacional de Metrología (CENAM), All-Russia D.I. Mendeleyev Scientific and Research Institute for Metrology (VNIIM), Australian Commonwealth Scientific and Research Organization (CSIRO), National Research Laboratory of Metrology (NRLM), Korea Research Institute of Standards and Science (KRISS), National Institute of Metrology (NIM).

[17] Salsbury James G, Inloes Michael (2006). In-situ Calibration of Temperature Compensations Systems Using Differential Length Measurements.

